# LightGBM-Based Human Action Recognition Using Sensors

**DOI:** 10.3390/s25123704

**Published:** 2025-06-13

**Authors:** Yinuo Liu, Ziwei Chen

**Affiliations:** 1 Department of Computer Science and Technology, College of Information Engineering, Northwest A&F University, Yangling, Xianyang 712100, China; liuyn@nwafu.edu.cn; 2 Department of Electronics, Beijing Jiaotong University, Beijing 100044, China

**Keywords:** human activity recognition, feature selection, LightGBM

## Abstract

In recent years, research on human activity recognition (HAR) on smartphones has received extensive attention due to its portability. However, the discrimination issues between similar activities such as leaning forward and walking forward, as well as going up and down stairs, are hard to deal with. This paper conducts HAR based on the sensors of smartphones, i.e., accelerometers and gyroscopes. First, a feature extraction method for sensor data from both the time domain and frequency domain is designed to obtain more than 300 features, aiming to enhance the accuracy and stability of recognition. Then, the LightGBM (version 4.5.0) algorithm is utilized to comprehensively analyze the above-mentioned extracted features, with the goal of improving the accuracy of similar activity recognition. Through simulation experiments, it is demonstrated that the feature extraction method proposed in this paper has improved the accuracy of HAR. Compared with classical machine learning algorithms such as random forest (version 1.5.2) and XGBoost (version 2.1.3), the LightGBM algorithm shows improved performance in terms of the accuracy rate, which reaches 94.98%. Moreover, after searching for the model parameters using grid search, the prediction accuracy of LightGBM can be increased to 95.35%. Finally, using feature selection and dimensionality reduction, the efficiency of the model is further improved, achieving a 70.14% increase in time efficiency without reducing the accuracy rate.

## 1. Introduction

With the popularization of the construction of an intelligent living environment and Internet of Things (IoT) devices, human activity recognition is considered to be of great significance in assisting the research on interactive environments and devices. By analyzing and identifying human activities and assigning corresponding labels, multi-dimensional data in various scenarios can be classified. The processed behavior labels assist in achieving various, more refined functions. At present, the application fields of human activity recognition (HAR) cover many fields, such as smart homes [1], health monitoring [2], indoor navigation [3], security monitoring systems [4], and other fields [5]. HAR technology is typically implemented by a variety of mechanisms, including computer vision [6], WiFi signals [7], wearable sensors [8], and portable mobile devices such as smartphones [9] and smartwatches [10]. Computer vision for behavior judgment focuses on the use of cameras for recognition, but this method is inconvenient to implement due to the limited locations of camera installation and the easy leakage of user privacy and security issues. Moreover, WiFi is more restricted indoors, so it is not easy to use for real-time behavior monitoring of users. In addition, wearable devices (such as smart bracelets and smart watches) require users to actively wear them, which may affect the frequency of use due to forgetfulness or discomfort. In comparison, portable mobile devices such as smartphones are daily necessities that users can carry around without having to wear or carry other devices to complete activity recognition tasks, which generate real-time behavioral data through sensors such as accelerometers and gyroscopes, and are more suitable for aiding judgment of human activities.

In recent years, remarkable progress has been made in the research of HAR based on smartphone sensors, with application fields covering many aspects. In the context of health monitoring and chronic diseases [11], smartphone sensors are used to identify users’ daily activities to monitor their health status, especially in chronic disease interventions. In terms of emotion recognition and mental health [12], by analyzing the behavior patterns and activities of users carrying mobile phones, their emotional state is identified, and then mental health support is provided. Additionally, in terms of interaction in smart home environments [13], smartphones recognize the user’s behavior to realize the automatic control of smart home devices and provide convenience in the user’s life.

Various methods are used for HAR, including machine learning algorithms such as support vector machines (SVMs) [14], random forests [15], and k-nearest neighbors (KNNs) [16]. At the same time, deep learning models are gradually being applied in this field, and common methods include convolutional neural networks (CNNs) [17], long short-term memory networks (LSTMs) [18], and their combined models [19]. Current research on human activity recognition focuses on deep learning and machine learning methods, and the main difficulty of most machine learning algorithms is that the process of feature extraction [20] and feature selection [21] is time-consuming. The redundant and less important data in the available feature set increase the calculation time of model training, while the presence of noise also reduces training performance. CNNs can extract spatial features, while LSTMs can extract time series features, and both CNNs and recurrent neural networks (RNNs) have single-feature extraction capabilities. Although machine learning and deep learning have achieved certain results in feature extraction, most current studies use frequency domain features such as Fourier transform and discrete cosine transform to classify and identify activities. Instability problems are prone to occur when identifying similar activities [22]. For example, the recognition accuracy of sitting and lying movements is not high, and the recognition accuracy that is needed to distinguish directions and going up and down stairs is also slightly reduced. In addition, although LSTMs can directly process time series data, they lack the inductive understanding of time series data statistics and the extraction of features from a small number of training sets.

In order to solve the above problems, this paper models the HAR problem based on LightGBM, adds relevant time domain and frequency domain features to the feature selection to improve the robustness of the model to different types of data, optimizes the recognition model by adjusting the hyperparameters, verifies the effect of the proposed method in improving the prediction accuracy by comparing it with the traditional machine learning algorithm, and finally, in order to improve the efficiency of the model, the feature importance of the model is analyzed and the feature quantity is simplified to achieve a shorter training time and prediction time while maintaining a certain model prediction rate. Finally, the next steps of research are summarized, along with the prediction of various activities.

This article is structured as follows. Section 2 introduces the relevant work, Section 3 describes the dataset, data preprocessing, feature extraction, and other aspects in detail, and designs the LightGBM-based HAR algorithm based on the above process, and Section 4 is the simulation analysis, which expounds on the algorithm improvement, including the processing of features, discusses the prediction of each activity, and then compares the classification methods of different machine learning, as well as the parameter tuning and efficiency optimization of the algorithm. Finally, this paper summarizes the direction for the future.

## 2. Related Work

For quite some time, a variety of methods have been used to design human activity recognition algorithms from different perspectives, and have made remarkable achievements in training efficiency and prediction accuracy. Usually, the whole process of human activity recognition involves several important processes, such as data collection, data preprocessing, feature selection, and algorithm design [23].

In order to improve the accuracy of activity recognition, the range of collected features can be expanded in the initial data collection phase. Shumei Zhang et al. added frequency identification (RFID) and wireless sensor networks (WSNs) to the body sensor network, added location and surrounding environment attributes, and used an integrated multi-context-based fall detection algorithm to improve the accuracy of detecting certain fall behaviors [24].

In order to improve the recognition efficiency of dynamic and static similar motion situations, many scholars have conducted research on pre-segmentation of the phone’s dataset. Fen Miao et al. set up light and proximity sensors and status listeners to pre-classify the detected states, enhancing the flexibility of mobile phone placement and reducing the complexity of distinguishing activities [25]. However, in the classification process, the classification of continuous activity transitions and activities that need to be sensitive to direction, such as going up and down stairs, is not accurate. Therefore, in terms of recognition behavior switching, Qin Zou et al. [26] proposed an effective and seamless combination of a Deep Evolutionary Neural Network (DCNN) and Deep Recurrent Neural Network (DRNN), which process spatial domain signals and time series, respectively, to determine whether users are in a walking state, and divide them into walking sessions and non-walking sessions, which solves the problem of traditional methods not achieving high performance in inertia-based gait recognition. In terms of sedentary monitoring, Muhammad Fahim et al. [27] proposed to mine the situation in real time and assign contextual labels during the identification phase. The extracted feature vectors are fed to the classifier for classification. In the first stage, the accelerometer data is classified as either “active” or “stationary”. In the second stage, the classifier classifies the audio data.

The main advantage of using smartphones for human activity recognition is the portability of the phone, which also means that data collection is relatively easy. It is crucial to select and simplify the collected features in order to reduce the training time of the model and improve the updating efficiency of the model, so as to achieve the effect of the model quickly adapting to the accurate classification of different user groups’ behaviors. Junhua Ye et al. [28] used the Fourier transform, continuous cosine transform, wavelet transform, and other frequency domain processing methods to compare seven machine learning methods and create two deep learning methods, which improved the recognition accuracy of harsh environments and complex motions. Masud Ahmed et al. [29] introduced smartphone orientation constraints based on amplitude and agitation features, separated the genetic algorithm components from the filtered accelerometer raw data, and studied multiple filtering parameters to reduce waveform delay. M. M. Manca et al. [30] used dimensionality reduction techniques to identify and retain the most informative and predictive features for activity recognition to simplify the model and improve efficiency, and evaluated how effective different feature selection algorithms were in identifying the most predictive features. Karam Kumar Sahoo et al. [31] converted the accelerometer and gyroscope sensor data into spectral spectra, and then used a wrapper optimizer algorithm based on a combination of transfer learning models and a binary bat algorithm (BBA) to select the best features and classify the activities by the subset of the best features, which reduced the training time and improved the final classification performance.

In the past decade, human activity recognition using machine learning methods has been continuously developed, and LightGBM is a very promising solution for HAR, which could make human behavior analysis faster and more objective. Csizmadia Gábor et al. [32] used wearable smartwatches to collect information on children’s various play and daily activities, and successfully identified 17 out of 40 activities with AUC values above 0.8. The proposed model by Topuz, E. K. et al. [33] combines the balanced optimizer as an optimizer with the LightGBM classifier to address the problem of higher requirements of computing power and resources in devices with limited power and memory. In order to improve the recognition accuracy of similar actions, Junjie Zhang et al. [22] integrated residual structure and layer normalization into a bidirectional long short-term memory network. This integration enhanced the feature extraction capability of the network, and introduced an attention mechanism to optimize the final feature information. In the study of Morsheda Akter et al. [34], they integrated feature combinations from multiple convolution stages and also introduced an attention mechanism to extract finer features and generate more comprehensive feature representations, further improving the accuracy of the model. Prabhat Kumar et al. [35] used activity sensor data availability as contextual information. The proposed Deep Context Model (DCM) consists of a network of convolution and recursion to discover relevant activity patterns in a dual context. Convolutional networks are used for feature extraction in static contexts, and recurrent networks are used to memorize patterns in dynamic contexts.

## 3. LightGBM-Based HAR Algorithms

**Figure 1:**
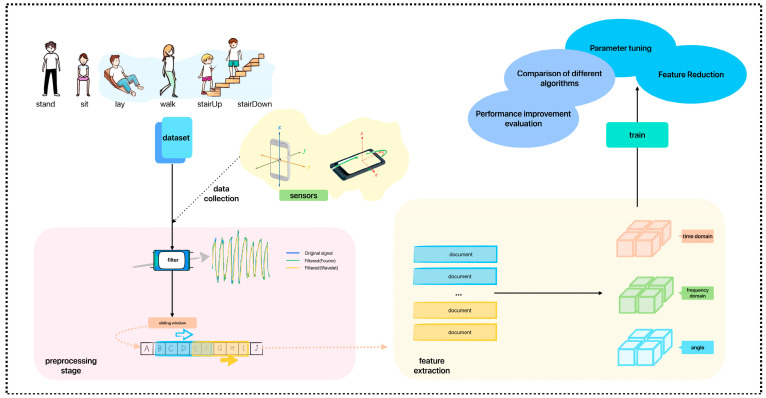
Algorithm block diagram.

To improve recognition accuracy for similar activities and optimize feature selection, this paper proposes a LightGBM-based HAR algorithm. Figure 1 is the algorithm block diagram. Data collection involves using smartphone sensor data from accelerometers and gyroscopes in the x, y, and z axes to identify six human activities: standing, sitting, lying, walking, and going up and down stairs. In the preprocessing stage, wavelet transform is used for filtering and noise reduction to process non-stationary signals. After filtering, signal segmentation is performed using a sliding window approach, defining the window size, step size (number of samples per move), and the initial sample shift. Data is extracted by specifying the window length, and multi-dimensional features are extracted from each window, such as the angle between the accelerometer and gyroscope axes, kurtosis, and autoregressive coefficients. Finally, the processed data is used to train various classification models to identify the best classifier.

### 3.1. Original Datasets

Researchers [33] have conducted experiments on UCI-HAR [36] and WISDM [37] datasets based on LightGBM for human activity recognition. This experiment will use the KU-HAR dataset [38] to test LightGBM on more angles and poses, which is also widely used in HAR research. It contained information on 18 different activities collected from 90 participants (75 men and 15 women) using smartphone sensors (the accelerometer and gyroscope). It collected 1945 original activity samples directly from participants and extracted 20,750 subsamples from them. The activities include six behavior patterns, as shown in Table 1: standing, sitting, lying, walking, going upstairs and going downstairs.

The dataset authors took into account the time delay between the start of recording and the start of activity, and due to this mismatch, the data collected in the first few seconds might not be from the corresponding activity at all. The same thing can also happen at the end of the sample, so a trimming operation is performed on the affected signal to remove these parts. This raw data prunes unnecessary parts of the sample (from start to tail only). Samples are interpolated to maintain a constant sampling rate of 100 Hz.

### 3.2. Preprocessing Stage

Before feature extraction, signal noise reduction is performed mainly using wavelet transform. The input data is wavelet denoised, decomposing the signal into multiple sub-bands, each containing different frequency information. Each wavelet coefficient is processed by calculating its maximum value and applying soft thresholding: if the absolute value is below the threshold, it is set to zero; otherwise, the threshold is subtracted. This method effectively removes noise while preserving the signal’s main characteristics. Finally, the inverse wavelet transform reconstructs the denoised signal.

The sliding window technique simplifies complex calculations into localized operations. The original time series data is divided into windows, where each can be smoothed through averaging to reduce noise and outliers. Additionally, sliding windows are used for time series prediction by segmenting data to forecast future trends, such as extracting autoregressive coefficients based on time series changes in this experiment.

### 3.3. Feature Extraction

Most of the previous related studies focused on the processing of frequency domain features. In this study, we obtained and calculated the frequency domain, time domain, and angle features from the accelerometer and gyroscope sensors to construct an effective feature vector to improve the recognition ability.

#### 3.3.1. Frequency Domain Characteristics

Frequency domain feature extraction includes short-time Fourier transform (STFT), discrete cosine transform (DCT), discrete wavelet transform (DWT), and features based on the Wigner–Ville distribution [21]. Frequency domain entropy measures the uncertainty or complexity of a signal; higher entropy indicates more complexity and potential information. STFT features (e.g., mean and standard deviation) capture time and frequency changes, allowing analysis of instantaneous frequency characteristics and modeling dynamic changes, making it suitable for non-stationary signals. DCT helps identify the main components of the signal, reducing redundant information and improving processing efficiency. DWT analyzes signals at different scales, capturing transient features and local changes, and extracting multi-resolution features by calculating the mean and standard deviation at various levels. Features from the Wigner–Ville distribution also aid in understanding instantaneous frequency characteristics and are useful for detecting modulation and transient behavior, as shown in Table 2.

For example, the acceleration direction feature vector generated by the fast Fourier transform algorithm and the frequency domain feature vector generated by the discrete cosine [27] and wavelet transforms are used in machine learning for sedentary identification. Compared with some machine learning algorithms such as SVM, it has poor interpretability, and it is more difficult to understand the basis of model decisions.

In the feature extraction part, the accelerometer signal is first denoised by wavelet transform, and then the input signal is subjected to fast Fourier transform to obtain the complex value of the frequency domain and the corresponding frequency. Next, the frequency range is divided into bands of equal width, the energy of each band is obtained by the square sum of the FFT results in the band calculated by Equations (1) and (2), and then the energy of multiple bands is calculated.

(1) $$E_{i}=\sum_{f\in {Band}_{i}}|X(f){|}^{2}$$

(2) $$|X(f)|=\sqrt{Re(X(f){)}^{2}+Im(X(f){)}^{2\;\;\;\;\;\;}}$$

where *X*(*f*) is the complex value of the frequency domain signal obtained through FFT. *X*(*f*) is the amplitude (modulus) of the frequency domain signal, representing the signal strength at frequency f. Band represents the frequency range of the i-th frequency band, usually achieved by dividing the entire frequency range into multiple equally wide frequency bands.

#### 3.3.2. Time Domain Characteristics

In the time domain feature extraction stage, the extracted features can be roughly divided into the following categories: basic statistical features, such as mean, standard deviation, median absolute deviation, etc.; distribution characteristics, such as skewness, kurtosis, etc.; signal energy characteristics; autoregressive features; and other features.

In addition to the characteristic values and basic features in the frequency domain, some time domain features are added in this experiment, and the autoregressive coefficient (autocorrelation of time series data) is mainly used in this experiment. An autoregressive model is a statistical model that predicts future values by observing the historical values of a certain time series. The autoregressive coefficient is a weighted sum of subsequent values that depend primarily on the values of the previous time points. Autoregression can be used to capture trends and periodicity in time series data, which is of great significance for modeling dynamic properties in different behavioral states. Please refer to Table 3 for the characteristics of the time domain.

#### 3.3.3. Angle Features

Our experiment also incorporates features related to angles, as shown in Table 4. Calculating the angle between different axes reflects the relative direction change of the signal, which helps to understand the relationship between different sensor readings, and has obvious advantages for the identification of going up and down stairs. It can not only be used for basic statistical analysis, but also as an input to the model, so as to realize motion recognition, detection, activity classification, etc. Through these features, sensing data can be more comprehensively understood and used, and the accuracy of data analysis can be improved.

### 3.4. LightGBM-Based HAR Method

As an efficient gradient boosting decision tree framework, LightGBM has the characteristics of fast speed, low memory consumption, and high accuracy, which is very suitable for processing large-scale datasets.

LightGBM uses a histogram algorithm to build a decision tree. The algorithm discretizes the continuous floating-point features into k integers and constructs a histogram with a width of k. When traversing the data, the statistics are accumulated in the histogram according to the discretized values as indexes, and then the optimal segmentation points are found according to the discrete values of the histogram. The Gradient-based One-Side Sampling algorithm reduces the amount of computation by retaining data samples with large gradients and randomly sampling data samples with small gradients, thereby improving the training speed. The Exclusive Feature Bundling algorithm accelerates model training by bundling mutually exclusive features together and reducing the dimensionality of features. The leaf-wise algorithm selects the leaf with the largest splitting gain each time for splitting, and the leaf-wise algorithm can reduce the error more effectively than the layer-wise grow-by-layer algorithm, but the maximum depth limit needs to be set to prevent overfitting.

The KU-HAR dataset was selected for the experiment, which includes six activity modes: sitting, standing, lying, walking, going upstairs, and going downstairs. It contains three-axis data from accelerometers and gyroscopes, along with timestamps. First, the collected three-axis acceleration and angular velocity data are denoised using discrete wavelet transform to improve signal quality. Then, multi-level features are extracted, including time domain statistical features, autoregressive coefficients, and frequency domain features, and the angles between different axes are calculated to quantify the spatial direction relationship. The data is processed in segments using a sliding window to enhance the ability to capture time series dynamics. LightGBM classifies and recognizes six types of human activities. The processed dataset contains 924 dimensional features and corresponding labels. After reading, the features are checked for duplicate, constant, and low-variance features to ensure data quality. Random partitioning is used to divide the data into training and test sets with a ratio of about 70%:30%, and the feature scale is unified through standardization. Model setting parameters such as the number of leaf nodes, maximum tree depth, learning rate, and number of basic learners are used to balance the training effect and time overhead. The time consumed for model training and prediction is recorded during training to ensure the timeliness evaluation of the experiment. A 10-fold cross-validation is performed during training to robustly evaluate the model performance. In the evaluation phase, the accuracy, confusion matrix, and classification report (including precision, recall, and F1-score) are calculated on the test set, and feature importance analysis is performed to evaluate the contribution of each feature to the prediction. Hyperparameters have a significant impact on machine learning algorithms, so grid search is used to optimize them. During the tuning process, the target model, hyperparameter grid, scoring criteria, and cross-validation folds are defined. Grid search evaluates all hyperparameter combinations and determines the best settings through cross-validation. Finally, the performance of the model is evaluated on the test set to verify the effect of hyperparameter adjustment.

## 4. Simulation and Analysis

In this section, we will introduce the evaluation indicators of the experimental results, as well as the improvement of the existing human activity recognition algorithms, including feature extraction, parameter setting, and time optimization, and will also use the same features to compare the performance of different algorithms, and will finally analyze the classification of the six human activities identified by the proposed algorithm.

### 4.1. Evaluation Indicators

The performance of the human activity recognition model proposed in this study was evaluated using the precision, recall, F1-score, and 10 cross-validation evaluation indicators. The dataset is randomly divided, with about 30% of the samples used as the test set and the rest as the training set. This random division ensures that the test data is independent of the training data and has a consistent distribution, which helps to objectively evaluate the generalization ability of the model. The division ratio was adjusted through multiple experiments, and finally the division parameters that could achieve the best classification performance were selected.

The experiment used a sliding window method in the signal segmentation phase. The window size and step size (i.e., the number of samples per move) are first defined, followed by the number of samples each window contains (in this case, 100 data points), and the number of samples that move forward at the start of the window each time the window is moved (in this case, 50 data points). In the time period selection stage of the autoregressive coefficients in the feature extraction stage, the top 10 data points were selected to predict the current data.

### 4.2. Algorithm Improvements

#### 4.2.1. Performance Gain by Feature Selection

Table 5 compares the classification results using the features of this experiment with the features commonly used in feature extraction in existing experiments (such as short-time Fourier transform, discrete cosine transform, etc.). The dimension difference is eliminated through standardization, and then the key features are screened. The support vector machine (SVM) is then used to build a classification model, and the generalization performance is evaluated through 10-fold cross-validation, and the accuracy is verified on an independent test set. The cross-validation score variance is 6.96 × 10^−5^, and the cross-validation score standard deviation is 0.0083.

**Figure 2:**
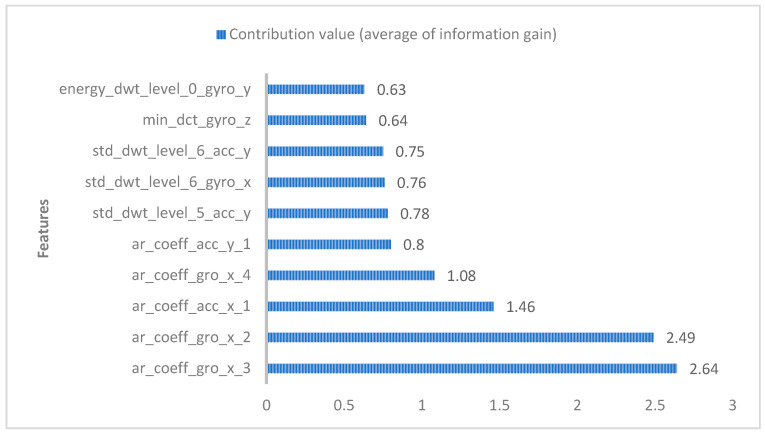
Feature contributions.

The features presented in this paper contribute by a large proportion to the classification, and the main features used in the experiment are shown in Figure 2. In terms of the time domain, it can be seen that the two parameters of the autoregressive coefficient of the gyroscope on the x-axis and the autoregressive coefficient of the accelerometer on the x-axis account for 75.95% of the contribution to human activity recognition, and the top five contributions to the classification of human activities are the time domain features added in this paper. In the frequency domain, the discrete wavelet transform at the sixth–eighth positions and the discrete cosine transform at the ninth gyroscope in the z-axis contribute the largest value. By adding the angle-related features, such as the angle between the x-axis and y-axis of the accelerometer, the average prediction accuracy generated is 94.29% compared with the average prediction accuracy of the X, Y, and Z axes using accelerometers and gyroscopes, which is higher than the average prediction rate of 92.99% without angle features, and 1.3% higher than the average prediction accuracy of the angle features, as shown in the Table 6.

The autoregressive coefficients (ar_coeff), as time domain features, contribute the most to the classification of human activities in the features, as shown in Figure 2. Different human activities (e.g., sitting, standing, lying, walking, and going up and down stairs) are unique in the performance of gyroscopes and accelerometers. For example, in the static positions of sitting or lying, the value of the gyroscope changes less, and the autoregressive coefficient may be lower; however, in the standing state, the autoregressive coefficient fluctuates slightly, but the change is not as obvious as that in the exercise state. When walking, the x-axis reading of the gyroscope fluctuates significantly because the body swings from side to side, resulting in the autoregressive coefficient exhibiting significant periodicity. When going up and down stairs, gyroscopes and accelerometers produce noticeable amplitude changes, especially in the accelerometer values, that appear as pulsed fluctuations.

#### 4.2.2. Classification Results for Each Activity

Analyzing the classification results is crucial for evaluating the effectiveness of algorithms and improving models, as it can reveal the recognition precision of different behavioral labels, thereby guiding subsequent optimization directions. This paragraph mainly compares the impact of different feature extraction methods on the classification results, using SVM. Table 7, Table 8, Table 9 and Table 10 are the results of the classification of each label by the existing algorithm and the experimental algorithm, and it can be seen that the recognition precision of sitting, standing, and lying down has been greatly improved. In terms of precision, the three types of behaviors “stand”, “sit”, and “lay” improved by about 6%, 10%, and 8%, respectively. The autoregressive model in this experimental algorithm can effectively capture the time dependence in the sequence data, which means that it can identify the relationship between the behavior state at one moment and the previous moment, which is especially important for the recognition of static behaviors such as sitting, standing, and lying.

### 4.3. Classification Outcome Assessment

In order to verify the advantages of the proposed algorithm, this paper uses traditional machine learning and deep learning methods as a comparison. Table 11 shows the results of different algorithms classifying six human behaviors by processing the same feature data. The average cross-validation score of each algorithm shows the overall performance of the model, and the higher the value, the better the generalization ability of the model. Here, LightGBM (95.19%) performs best, followed by XGBoost (95.16%), while SVM (93.74%) and LSTM (93.56%) score slightly lower.

Among the above four machine learning algorithms, the classification effects of LightGBM and XGBoost are better than those of SVM and RF, and the 10-fold cross-validation result reaches about 95%. XGBoost performs well with unbalanced data, handles missing values effectively, and has strong regularization capabilities to prevent overfitting. LightGBM is suitable for large-scale data, with a fast training speed and low memory footprint, and supports direct processing of class features. High-dimensional sparse data can be processed. SVM performs well in high-dimensional spaces, especially for linearly separable data, using different kernel functions to deal with nonlinear problems. RF is relatively less accurate because it is not as adaptable to high-dimensional sparse data as other algorithms. For deep learning methods, CNN has balanced performance in static and dynamic activity recognition, but its overall performance is slightly lower, which may be limited by its insufficient modeling ability of long-term temporal dependencies and the need for larger datasets. Compared with CNN, LSTM is better at capturing dynamic changes in time, and therefore performs better in identifying dynamic activities (such as walking and going up and down stairs), but its ability to distinguish static activities (such as sitting, standing, and lying down) may be similar.

### 4.4. Parameter Tuning

**Figure 3:**
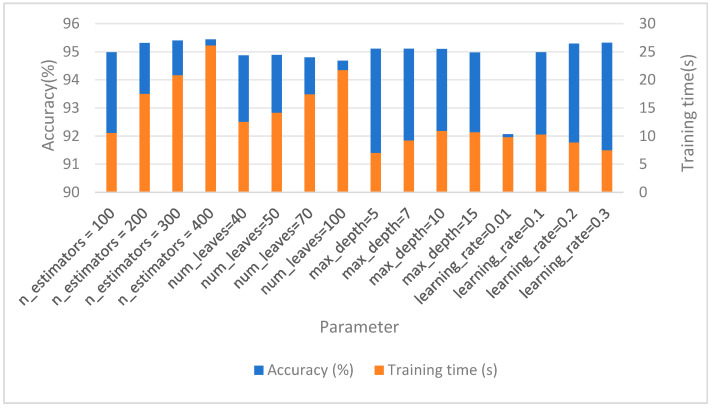
LightGBM hyperparameter optimization.

In the parameter tuning stage, a large number of parameter adjustment experiments were carried out on LightGBM and XGBoost. We divided the training and validation set and the testing set to ensure that the testing set did not participate in training and parameter tuning, effectively avoiding the risk of data leakage. At the same time, we used the validation set to optimize the model. “n_estimators” indicates the number of base learners, “max_depth” indicates the maximum depth of the tree, “learning_rate” indicates the learning rate, and “num_leaves” indicates the number of leaf nodes. Finally, LightGBM was the best when the number of base learners was 400, and the average prediction accuracy increased from 95.19% to 95.44% before parameter tuning, which was better than the test results of XGBoost (95.16%). Table 12 and Figure 3 show the results of LightGBM hyperparameter optimization. Increasing the number of base learners often improves the expressiveness of the model. The choice of 400 base learners allows the model to learn more complex patterns and features in the data, thereby improving classification accuracy. The “max_depth” controls the depth of each decision tree, which affects the complexity of the model. Proper tree depth can capture the complexity of the data, but trees that are too deep can lead to overfitting. At the same time, the size range of the parameters shown should be adjusted so that the training time is not too long, and finally, the accuracy of the model is increased to 95.44% when the number of base learners is 400.

### 4.5. Algorithm Efficiency Optimization

A total of 924 features were used for training in this experiment, and the prediction accuracy of the four behaviors of sitting, standing, lying, and walking was significantly improved. However, considering the real-time nature of human activity recognition with smartphones, the average model training efficiency needs to be improved so that the model can be updated more quickly.

**Figure 4:**
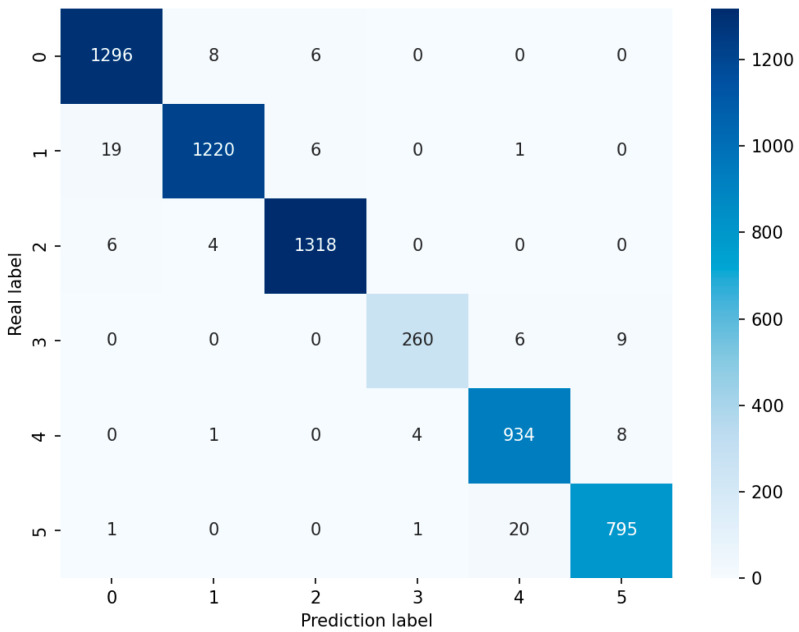
Confusion matrix.

Through the contribution values of each feature (as shown in Figure 2) and the heatmap (showing the correlation between different features), we selected the features with high importance to the prediction results, and filtered out highly correlated eigenvalue pairs to avoid using redundant features in model training. Ultimately, a total of 325 features were chosen for feature reduction, achieving the effect of improving the algorithm time efficiency. Through extensive testing and comparative experiments, the training results of LightGBM were more ideal than those of XGBoost, and the method used in this experiment achieved an average accuracy of 95.50%. Finally, the accuracy of the LightGBM classification results was evaluated through the confusion matrix, as shown in Figure 4.

Table 13 shows the average results of the six human behavior activities classified using LightGBM. The average run time is the average program running time, and the average training time is the model training time.

It can be seen that the time efficiency of the model is reduced by 70.14%, and the average model training time is reduced by 79.16%, without reducing the accuracy. By reducing the number of features, the complexity of the model is reduced, which makes the model easier to train and better generalized to new datasets, and reduces the degree of overfitting of the model due to noise or irrelevant features in the high-dimensional feature space.

## 5. Conclusions

Research based on smartphone sensors is a key part of the human activity recognition (HAR) field. This paper reviews previous studies on human activity recognition, then provides the methodology of this study and introduces the data preprocessing process, including data extraction from accelerometers and gyroscopes, denoising by Fourier transform and wavelet transform, and the design, extraction, and selection of time domain and frequency domain features. In addition, this paper describes the process of model training, compares classification metrics between different machine learning models, and discusses how to optimize the algorithm in terms of parameters and time.

In this study, four experiments were conducted using machine learning methods. The first is the feature improvement experiment. By adding autoregressive coefficients and other related time domain features, using SVM to classify six human activities, the accuracy is improved from 87.65% to 93.74%, and it can be seen that the recognition efficiency of the three similar activities of sitting, standing, and lying has been significantly improved. The second is to compare different machine learning classification methods. This paper uses 10×cross-validation for classification result evaluation, and it can be seen that the LightGBM and XGBoost models perform better, with results of 95.19% and 95.16%, respectively. The third is parameter tuning, and the experiment finds that LightGBM is the best when the number of base learners is 400, and the average prediction accuracy is increased from 95.19% to 95.44% before parameter tuning. The fourth is algorithm optimization; by performing feature reduction, the algorithm efficiency is improved in terms of time, and the LightGBM-based HAR algorithm achieved an average accuracy of 95.50% (after feature reduction).

In this study, the recognition accuracy of similar activities was significantly improved, and it was also found that the classification of the “upstairs” and “downstairs” activities using the features proposed in this experiment was better than the confusing results mentioned in the previous research literature. However, while the current work focuses on offline performance, future deployments must address challenges such as battery consumption and computational overhead on resource-constrained devices to ensure practical viability. In the future, multimodal data fusion, such as integrating smart phones with the Internet of Things interaction network where users are located and extracting interaction data, should be considered so as to better improve the prediction accuracy of user behavior.

## Figures and Tables

**Table 1:** Partial human activities in the KU-HAR dataset.

Activities	Record Duration
Stand➞ Standing still	1 min
Sit➞ Sitting still	1 min
Lay➞ Laying still	1 min
Walk➞ Walking 20 m	≈12 s
Stair-up➞ Ascending on a set of stairs	≈1 min
Stair-down➞ Descending from a set of stairs	≈50 s

**Table 2:** Frequency domain characteristics extracted from accelerometers and gyroscopes.

Features	Meaning
fd_entropy	The frequency domain entropy of a signal
band_energy	The signal spectrum is divided into frequency bands, and the energy of each band is calculated to identify the energy distribution within the frequency range.
stft_features	Short-time Fourier transform
dct_features	Discrete cosine transform
dwt_features	Discrete wavelet transforms
wigner_distribution_features	The Wigner–Ville distribution

**Table 3:** Time domain characteristics extracted from accelerometers and gyroscopes.

Feature	Meaning
mean	The average value of the signal on the corresponding axis (x, y, or z).
std	Standard deviation, which reflects how volatile the signal is.
mad	Median absolute deviation, which represents the median of the absolute difference from the median for each data point in the dataset.
energy	The average of the sum of squares of the signal.
skewness	Skewness, which reflects the asymmetry of the distribution.
max	The maximum value of the signal on the corresponding axis.
min	The minimum value of the signal on the corresponding axis.
iqr	The interquartile distance of the signal on the corresponding axis indicates how much the data is dispersed.
entropy	The entropy of the signal on the corresponding axis, which measures the uncertainty of the signal.
ar_coeff	The autoregressive coefficient of the signal on the corresponding axis, which is used to describe the linear relationship of the signal.
kurtosis	Kurtosis, which reflects how sharp the signal distribution is.
meanFreq	The weighted average frequency of the signal on the corresponding axis represents the frequency distribution of the signal.
maxInds	The indexed position of the maximum value of the signal on the corresponding axis.
median	The median of the signal on the corresponding axis.
rms	The root mean square value of the signal on the corresponding axis, reflecting the RMS value of the signal.
percentile_75	The 75th percentile of the signal on the corresponding axis, indicating the high-end distribution of the signal.
variance	The variance of the signal on the corresponding axis, which indicates how discrete the signal is.
binned_distribution	The binning probability distribution of the signal on the corresponding axis indicates the distribution of the signal in different intervals.
zcr	The zero crossover rate of the signal on the corresponding axis, which indicates the frequency of the signal change.

**Table 4:** Angle features.

Feature	Meaning
angle_xy/angle_xz/angle_yz	The angle of the accelerometer between the *x*-axis and *y*-axis/*x*-axis and *z*-axis/*y*-axis and *z*-axis.
angle_gyro_xy/angle_gyro_xz/angle_gyro_xz	The angle of the gyroscope between the *x*-axis and *y*-axis/*x*-axis and *z*-axis/*y*-axis and *z*-axis.

**Table 5:** SVM simulation results.

Methods	Accuracy (%)
Previous methods	87.65
Our method	93.74

**Table 6:** Contribution of angle-related features.

Methods	Average Prediction Accuracy (%)
Time domain + frequency domain	92.99
Time domain + frequency domain + angle	94.29

**Table 7:** Previous methods: Each human action.

Label	Precision	Recall	F1-Score
Stand	0.87	0.86	0.85
Sit	0.81	0.79	0.80
Lay	0.85	0.86	0.85
Walk	0.97	0.93	0.95
Upstairs	0.96	0.96	0.96
Downstairs	0.95	0.96	0.96

**Table 8:** Previous methods: Overall performance.

Evaluation	Precision	Recall	F1-Score	Accuracy
Macro avg	0.90	0.89	0.90	0.88
Weighted avg	0.88	0.88	0.88	

**Table 9:** Our methods: Each human action.

Human Action	Precision	Recall	F1-Score
Stand	0.93	0.93	0.93
Sit	0.91	0.91	0.91
Lay	0.93	0.93	0.93
Walk	0.98	0.95	0.96
Upstairs	0.96	0.96	0.96
Downstairs	0.96	0.96	0.96

**Table 10:** Our method: Overall performance.

Overall	Precision	Recall	F1-Score	Accuracy
Macro avg	0.95	0.94	0.94	0.94
Weighted avg	0.94	0.94	0.94	

**Table 11:** Cross-validation of the evaluation results 10-fold for different algorithms.

Models	Mean Cross-Validation Score (10-Fold)%	Precision	Recall	F1-Score
SVM	93.74	0.95	0.94	0.94
RF	91.95	0.97	0.96	0.96
LightGBM	95.19	0.98	0.97	0.98
XGBoost	95.16	0.98	0.97	0.98
CNN	92.19	0.92	0.93	0.93
LSTM	93.56	0.93	0.93	0.93

**Table 12:** LightGBM hyperparameter optimization.

Parameter	Parameter Values	Accuracy (%)	Training Time (s)
n_estimators	100	94.98	10.52
	200	95.31	17.5
	300	95.4	20.8
	400	95.44	26.09
num_leaves	40	94.87	12.51
	50	94.89	14.14
	70	94.8	17.42
	100	94.68	21.7
max_depth	5	95.11	6.99
	7	95.11	9.18
	10	95.1	10.87
	15	94.97	10.63
learning_rate	0.01	92.07	9.77
	0.1	94.98	10.25
	0.2	95.29	8.82
	0.3	95.32	7.45

**Table 13:** LightGBM classification efficiency optimization.

Index	Before Simplification	After Simplification
Average accuracy (%)	95.44	95.50
Average run time (s)	434.28	129.66
Average training time (s)	32.40	6.75
Forecast time (s)	0.13	0.06

## Data Availability

The raw data supporting the conclusions of this article will be made available by the authors on request.
